# Insights into the Hydration Layer of Reduced Graphene Oxides: A Computational Study

**DOI:** 10.1002/cssc.202400520

**Published:** 2024-11-17

**Authors:** Filippo Savazzi, Francesca Risplendi, Giancarlo Cicero

**Affiliations:** ^1^ Dipartimento di Scienza Applicata e Tecnologia Politecnico di Torino Corso Duca degli Abruzzi 24 10129 Torino Italy

**Keywords:** graphene oxide, interface, water, Density functional theory, ab initio molecular dynamics

## Abstract

Reduced graphene oxide (rGO) has emerged as a versatile material with diverse applications, particularly in aqueous environments. Understanding its interactions with water molecules is crucial for various fields, ranging from energy storage to sensing. In this study, we investigate the behavior of graphene and rGO in water, focusing on elucidating their wetting properties and the influence of oxygen‐containing functional groups. Through extensive molecular dynamics simulations, we analyze the orientation and electrostatic dipole of water molecules near the rGO interface, revealing a direct correlation between rGO hydrophilicity and oxidation level. Specifically, we observe stronger hydrogen bonding networks near higher coverage rGO monolayers, indicating enhanced hydrophilicity. Furthermore, by studying water confined between rGO layers, we find uniform water transport with lateral self‐diffusion coefficients comparable to bulk water, highlighting the potential of rGO membranes in various applications. Our findings provide insights into the atomic‐scale interactions governing rGO‐water interfaces, paving the way for the rational design of graphene‐based materials for application in aqueous environments.

## Graphical abstract

The hydration behavior of reduced graphene oxide (rGO) is studied using ab initio molecular dynamics simulations, revealing the role of oxygen‐containing functional groups in determining rGO′s wettability.

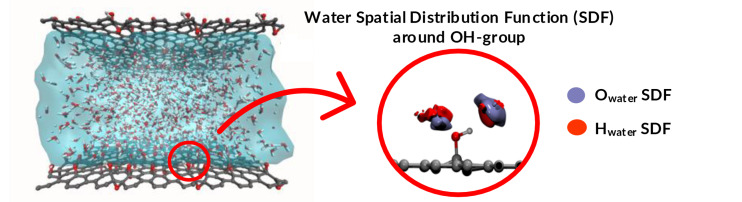

## Introduction

The importance of interfaces between water and graphitic surfaces, including their oxide counterparts like graphene oxide (GO) and reduced GO (rGO), has grown substantially across diverse fields such as water filtration, environmental remediation, energy storage, biomedical applications, and beyond. While GO is characterized by large content of oxygen‐containing functional groups, rGO is derived from GO through reduction processes, resulting in fewer oxygen terminations and partially restored graphitic structure.^[1]^

In biomedical applications, rGO materials exhibit substantial potential in drug delivery, biosensing, tissue engineering, and medical imaging. The interplay between water and rGO surfaces profoundly impacts the biocompatibility, stability and functionality of graphene‐based biomedical devices, thus influencing their efficacy and safety for clinical use.[^2^, ^3^, ^4^, ^5^] Additionally, rGO′s exceptional properties like high electrical conductivity and chemical stability are crucial for energy storage and conversion devices such as batteries, supercapacitors, and fuel cells.[^6^, ^7^, ^8^, ^9^, ^10^, ^11^, ^12^, ^13^, ^14^, ^15^, ^16^, ^17^, ^18^] Understanding the interface between rGO and water is essential for optimizing various processes like ion transport and redox reactions.[^19^, ^20^]

In environmental contexts, understanding the interface between water and rGO surfaces is paramount for optimizing processes such as pollutant removal and wastewater treatment.[^21^, ^22^, ^23^, ^24^, ^25^, ^26^, ^27^, ^28^, ^29^, ^30^] The interface between rGO and water holds immense potential for transforming filtration technologies, offering efficient solutions for improving water quality and addressing water scarcity.[^27^, ^31^, ^32^, ^33^] The ability to precisely control this interface facilitates the development of membranes with uniform pore sizes and 2D nanochannels, enhancing the efficiency of desalination processes.[^33^, ^34^, ^35^] Additionally, as this material is easy to fabricate and deposit, the presence of oxygen‐containing species allows to produce pores of uniform size in its structure,[^27^, ^28^, ^29^] making an ideal single‐layer membrane. Thus, comprehending and designing the interface between rGO and water not only opens avenues for advancing various technological applications but also holds promise for addressing pressing environmental and societal challenges. Moreover, rGO flakes can be stacked upon each other, forming 2D nanochannels that allow water to permeate while rejecting solutes.[^33^, ^34^, ^35^]

Despite their technological relevance across various applications mentioned previously, there remain several unknown aspects regarding GO‐water interfaces that require further investigation to fully leverage their potential for industrial applications. Whereas the behavior of water molecules in proximity of graphene has been extensively studied, showing the clear hydrophobic character of this interface,^[36]^ the wettability of rGO is still debated. Due to its variable stoichometry and different interactions between the oxygen‐containing species on its surface and surrounding water molecules, rGO has very heterogeneous wetting properties which must be carefully investigated considering samples with different oxygen content. Previous atomistic studies investigated the role of specific oxygen‐containing species in determining the wettability of rGO, by means of *ab initio* molecular dynamics (MD) simulations.^[37]^ Nonetheless these studies are based on simplified rGO models that do not consider the complex variability of its stoichometry. Moreover, the influence of GO on surrounding water molecules is still relatively unexplored, as most publications in literature only focus on providing estimations of water diffusivity near the interface, based on classical MD simulations.[^38^, ^39^, ^40^] As results from these works are sometimes incoherent, some predicting an increase of water mobility near the interface while others predict a mobility reduction, a rigorous investigation based on realistic models of rGO and accurate first‐principle simulations is much needed.

In this research work, our primary objective is to explore the behavior of graphene and monolayer rGO in aqueous environments. We place particular attention on understanding the intricate interactions between monolayer structures at different degree of oxidation and water molecules. Our aim is to uncover the effects of these interactions on the chemo‐physical properties of water near the interface. In particular, we will investigate how the composition of rGO influences its wettability and understand what kind of structural and chemical modifications are induced in this material by the presence of water. To this extent, we will provide references about different interactions strength between water molecules and specific oxygen‐containing species in rGO. The role of rGO composition on the chemo‐physical properties of its interface with water, was neglected in previous studies, where the authors typically considered rGO with fixed stoichiometry.[^35^, ^37^, ^41^] Moreover, we will discuss the effects of confinement between rGO layers on the chemo‐physical properties of water molecules, considering interlayer distances which are typically found in multilayer rGO membranes.^[42]^ Finally we will consider whether the presence of oxygen‐containing groups in rGO have an influence on the diffusivity of confined water molecules, in order to shine light on the water mass transport mechanism in multilayer rGO membranes. To this extent, we performed accurate *ab initio* MD and DFT simulations to model the evolution of this material in water under realistic conditions and therefore investigate the microscopic interactions that characterize such important interfaces.

## Method

To explore the behavior of rGO/water interfaces, we constructed several initial model structures representing rGO monolayers at different oxidation degree. For this purpose, we created 12.83 Å×12.35 Å graphene supercells containing 60 carbon atoms. Following the procedure reported in Ref. [43] epoxide (−O−) and hydroxyl (−OH) species were uniformly distributed over these layers at different atomic coverage, Θ, which represents the number of ‐O‐ and ‐OH groups relative to the total number of carbon atoms in the supercell. Namely we considered rGO systems with the following compositions: 10 % −O− and 0 % −OH; 0 % −O− and 10 % −OH; 20 % −O–10 % −OH; 10 % −O–20 % −OH. Additionally, we modeled pristine graphene without any oxygen functional groups for reference. To prevent spurious interactions between replicas, a vacuum layer of approximately 15 Å was added perpendicular to the monolayers. All structures were relaxed by means of plane waves based DFT as implemented in Quantum Espresso,^[44]^ employing the Perdew‐Burke‐Ernzerhof (PBE) functional for exchange and correlation^[45]^ and ultrasoft pseudopotentials.^[46]^ Electronic wavefunctions were expanded up to an energy cutoff =28 Ry, the Brillouin zone was sampled with a () Monkhorst‐Pack grid, while to relax atomic positions forces were minimized down to 26 meV/Å.

**Figure 1:**
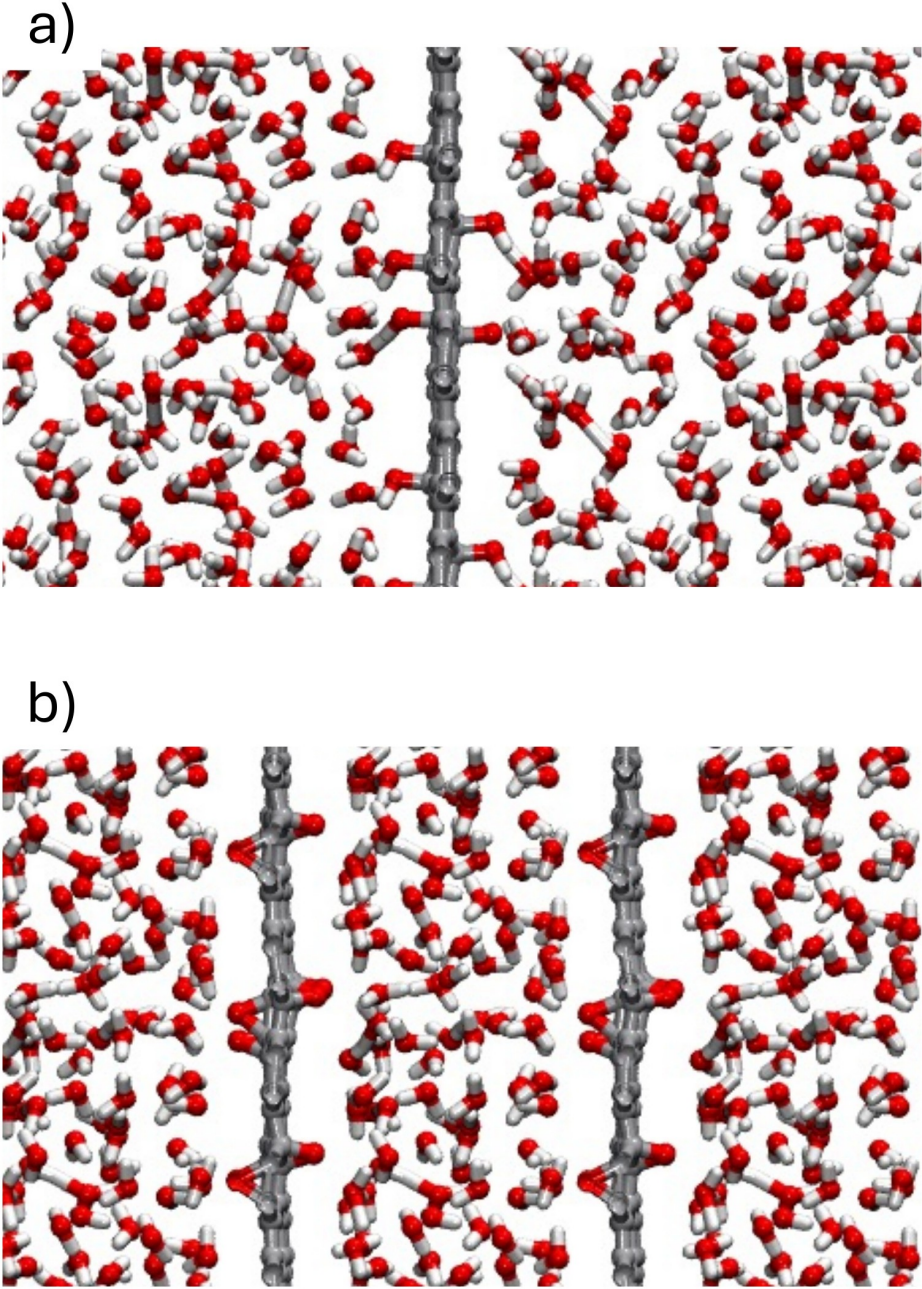
Model structures representing a single rGO monolayer dispersed in water (panel a) and a multilayer rGO system in aqueous environment (panel b). Red, grey and white spheres represent oxygen, carbon and hydrogen atoms respectively.

To simulate a single monolayer dispersed in water, we expanded the supercell dimension perpendicular to the rGO layer to 25 Å (considering Periodic Boundary Conditions), incorporating 108 water molecules to achieve a water density of within the simulation domain. The initial distribution of water molecules was obtained by using the “gmx solvate” function of GROMACS[47] to ensure the desired density (see panel a of Figure 1). A supercell dimension of 25 Å is enough to accurately describe bulk water properties away from the interface, based on a study of graphene in water from Cicero and collaborators.[36] Instead, to investigate water confined between multilayer rGO structures, we set a supercell size in the direction perpendicular to the monolayer to 13.7 Å  which corresponds to the interlayer distance reported for multilayer rGO. This interlayer distance was selected based on experimental evidence reported in the literature, which shows that the interlayer distance between flakes changes from approximately 6 Å when completely dry to about 14 Å when fully soaked in water.[42] Accordingly, the number of water molecules wetting our rGO system was fixed at 59 water molecules per supercell, which corresponds to a water density of approximately and a pressure close to ambient conditions.

We simulated the evolution of the multilayer rGO (with different stoichiometries) at room temperature by means of Car‐Parrinello MD (CPMD) simulations,^[48]^ implemented within the Quantum Espresso suite. We used PBE exchange and correlation functional and ultrasoft pseudopotentials.^[46]^ Although it is recognized that semi‐local GGA functionals like PBE may not fully capture van der Waals dispersion interactions, these effects are largely mitigated by the dominant role of hydrogen bonding in water‐rGO interactions. Furthermore, at finite temperatures, thermal effects and induced dipoles enhance the accuracy of PBE, as demonstrated in previous studies.[^49^, ^50^] Electronic wavefunctions were expanded up to an energy cutoff =28 Ry and the Brillouin Zone was only sampled at Γ. We set an electron fictitious mass =200 a.u. and a timestep =4 a.u. for Verlet integration of the equations of motion. In all MD simulations we substituted hydrogen atoms with deuterium atoms, to ensure total energy conservation for the choses integration timestep. For the sake of readability we will address D2O and −OD as H2O molecules and −OH groups in the following.

For each interface model, after the initial system setup and electronic energy minimization, we increased the temperature of our system from ~0 K to ~400 K by means of velocity rescaling and equilibrated the system for ~2 ps. Since the purpose of the present investigation was to compare the properties of confined water with those of bulk water, we considered the temperature (400 K) at which, for bulk water, DFT using PBE and Born‐Oppenheimer dynamics yields results in good agreement with experiment for structural and diffusion properties at ambient conditions.[36, 51–52] To this extent, we payed particular attention to have all different atomic species at similar temperatures (~400 K) and avoid the presence of hot or cold species that would impair the accuracy of the investigation. When the system temperature stabilized, after ~2 ps, we performed 20 ps of microcanonical (NVE) MD during which we collected data for trajectory analysis. Although temperature control was not applied during these 20 ps, in all cases the system temperature remained stable at ~400 K as set initially. For each possible rGO composition and interlayer distance we created 10 different structures and selected the one with lowest ground‐state energy as the most stable, before starting the MD run. We analyzed MD trajectories in terms of water mass density distribution, water molecules orientation and electrostatic dipole in order to investigate rGO surface wettability and the properties of surrounding water, as a function of the material composition. To evaluate the electrostatic dipole of water molecules, we selected snapshots each 1 ps along the 20 ps trajectory, and calculated the maximally localized Wannier functions, as detailed by Marzari and collaborators.[53]

Finally, we investigated water diffusivity in multilayer rGO structures with different oxidation degree, by computing the mean squared displacement of water molecules and employing the Einstein relation. This defines a proportionality between the mean squared displacement of particles and the observation time, provided that the latter is sufficiently long (ideally infinite):

(1) $$D=\frac{1}{2d}\lim_{x\to \infty }\frac{\left\langle {\left[r\left(t_{0}+t\right)-r\left(t_{0}\right)\right]}^{2}\right\rangle }{t}$$

where d is the dimensionality of the system, r is the position of particles and finally D is the average self‐diffusion coefficient of those particles in the atomic system. We averaged the mean squared displacement over all water molecules and over all time origins, as suggested by Keffer,[54] to improve statistical sampling. After obtaining the mean squared displacement and verifying that the diffusion regime was reached, the self‐diffusion coefficient is obtained performing a least square regression of the curve that it defines as a function of time, neglecting the early steps of the dynamics when particles’ motion is in a ballistic regime. The slope of this fitting represents the self‐diffusion coefficient, as defined in Eq. (1). For simulations of 20 ps duration, the error in the self‐diffusion constant is approximately 20 to 30 %, as reported in Refs. [51, 52].

## Results and Discussion

### rGO Monolayer Wettability

**Figure 2:**
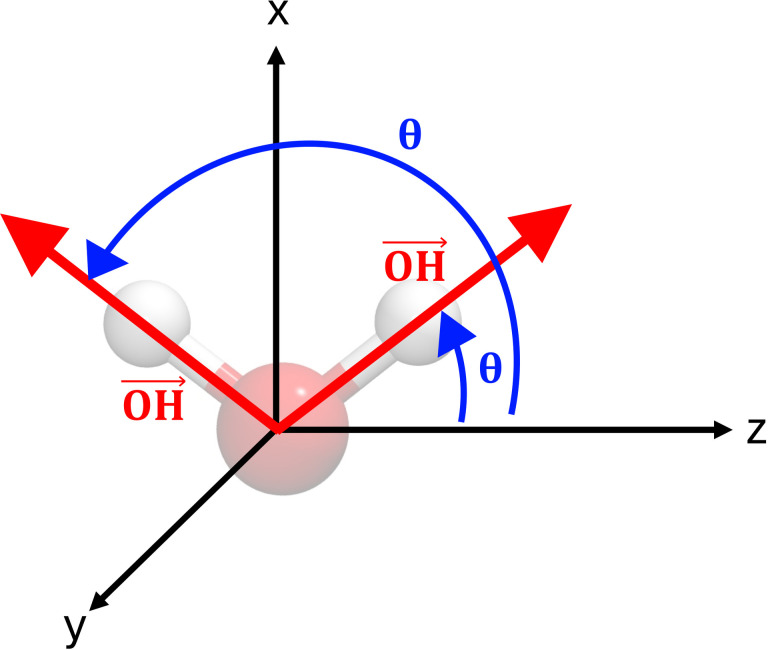
Definition of the O−H bond tilt angles *θ* formed by water bonds with respect to the direction perpendicular to the monolayer (z axis).

**Figure 3:**
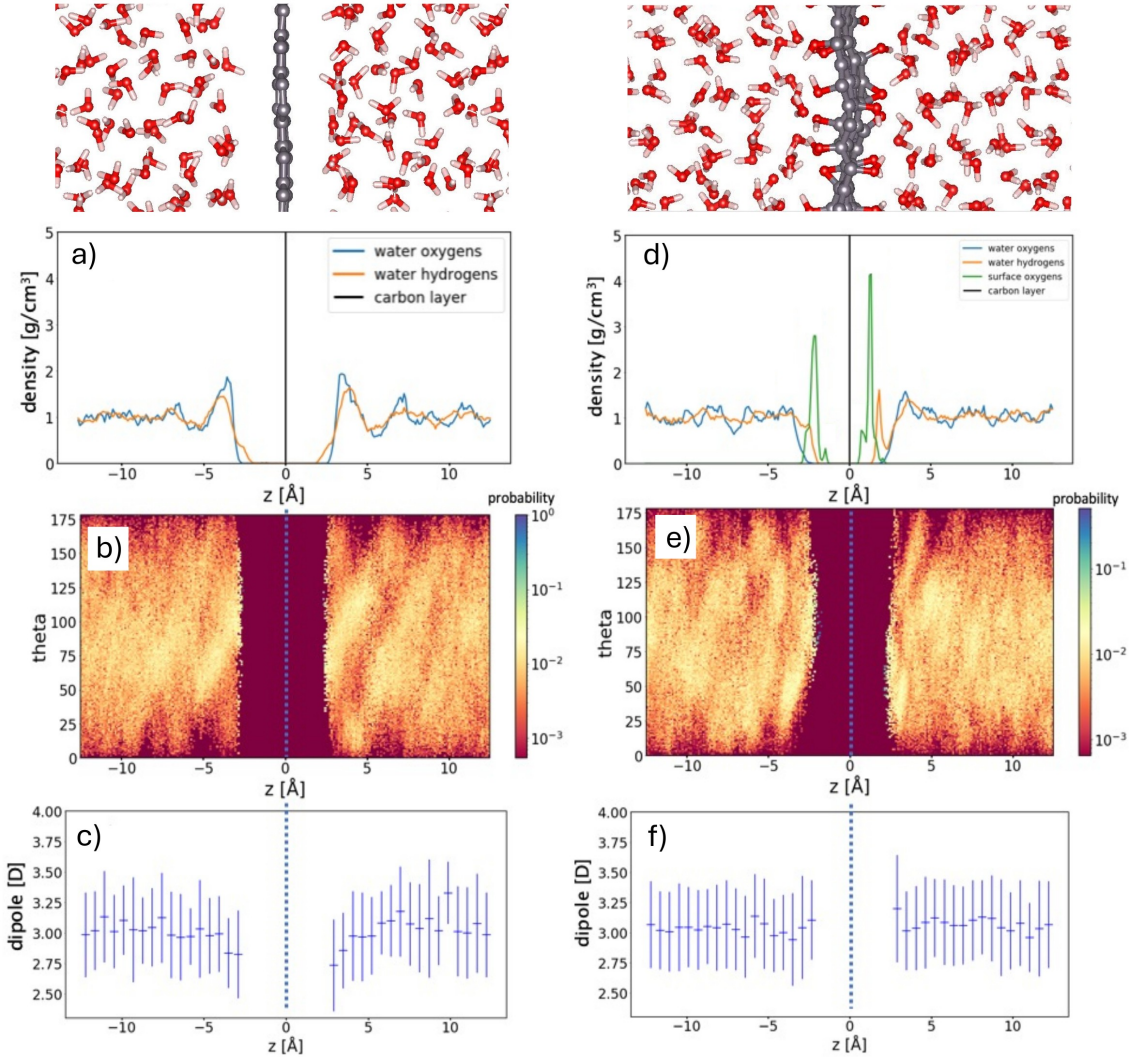
**Graphene‐water interface** (left panels). Mass density distribution of oxygens in water, as a function of their distance from the graphene layer (a). O−H bond orientation density distribution of H_2_O molecules (b). Average electric dipole distribution of H_2_O molecules (c). **rGO‐water interface (10 % −O−20 % −OH)** (right panels). Mass density distribution of oxygens in water and surface oxygen‐containing groups, as a function of their distance from the graphitic layer (d). O−H bond orientation density distribution of H_2_O molecules (e). Average electric dipole distribution of H_2_O molecules (f). in Both cases the supercell is 25 Å along the z direction, perpendicular to the interface.

In this study, we explored the wetting characteristics of rGO monolayers featuring varying concentrations of epoxides and hydroxyl groups. Our analysis focused on three key properties of water molecules within our interface models: the distribution of mass density, the orientation of water O−H bonds, and the average water electric dipoles in the interfacial layer. The analysis of water density perpendicular to the interface gives clear information about water structural properties, in particular about over‐structuring near the surface. We computed the density of water molecules as a function of the distance from graphene and averaged over all snapshots of the corresponding 20 ps MD trajectory. The orientation of O−H bond with respect to the direction perpendicular to the interface (see Figure 2 for the definitions of tilt angle, *θ*) instead, allows to identify variations of the preferential orientation of water molecules and further highlights modifications induced by the surface to the H‐bond network between H_2_O molecules.^[36]^ Finally, the average electric dipole distribution of water molecules provides an indication of the strength of the H‐bonds between H_2_O molecules, with lower dipole values suggesting a weakening of the H‐bond network. In Figure 3 panels a–c are reported the mass density distribution of carbon atoms and oxygens in water, the O−H bond orientation (vector) distribution and average electric dipole distribution of H2O molecules for the case of graphene dispersed in water. This interface has been extensively investigated in Ref. [36], which reported clear indications about the interactions between water and the hydrophobic graphene surface that we used to validate our method. From the mass density distribution of oxygen atoms in water molecules (Figure 3 a) it is possible to observe an exclusion layer, within 2.5 Å from the surface, where water does not penetrate, followed by a thin interfacial region where H2O molecules accumulate. It is clear that after 5 Å from the interface the effects of the latter on water density becomes negligible, with the recovery of a bulk‐like average density within 10 Å from the surface. High‐density peaks are located respectively at −3.5 Å and 3.5 Å from the graphene surface and reach a density (the graphene layer is placed in the origin of the z axis). This is in good agreement with results from,[36] where the authors found high‐density water peaks extending from 2.5 Å to 5 Å with maximum density . The O−H bond orientation density distribution of water (Figure 3b) shows how molecules near the interface are preferentially oriented with one hydrogen pointing toward graphene as highlighted by a higher probability of O−H bonds corresponding to 150°–170° near the right surface and 10°–20° near the left surface. Correspondingly the other −OH bonds are nearly parallel to the surface as shown by the high probability in correspondence of 120° near the left surface and 60° near the right surface.

Finally, we observed that the average electrostatic dipole distribution of interfacial H_2_O molecules (Figure 3c) is reduced to about 2.7 D–2.8 D near the surface, with respect to an average value of 3.1 D for water molecules in the bulk (farther than 5 Å from graphene), highlighting a weakening of the H‐bond network near the surface as suggested in Ref. [36]. This weakening of the H‐bond network formed by interfacial water molecules suggests that graphene is an hydrophobic material, as also previously reported in terms of a reduction of the number of H‐bonds formed by water molecules in proximity of a graphene layer.^[36]^ Moreover, the net orientation of interfacial H_2_O molecules with one O−H bond pointing toward the surface and the other lying parallel to it, suggests that they reorient to partially compensate the weakening of the H‐bond network by enhancing the interaction with neighboring molecules.

The interface between water and monolayer rGO is more complex than in the case of graphene, due to the presence of oxygen‐containing groups that interact with water and evolve in time. Our observations reveal transformations of −OH groups into negatively charged O^−^ species upon deprotonation. Additionally, we observed O^−^ species resulting also from the opening of the epoxide ring, although this occurrence appeared less frequent than −OH deprotonation. Finally, sporadic instances of −OH group desorption from rGO samples were detected, with subsequent diffusion within the interfacial layers of water. The analysis of samples with different stoichometries gave us clear indications that the presence of oxygen‐containing species results in rGO being a hydrophilic material. Nonetheless, the hydrophilicity of rGO is subject to important variations when its stoichiometry and oxidation level change, which themselves vary when interacting with water.

Regarding the restructuring of water at the surface of rGO, it is possible to identify general trends. If there are only epoxy groups on the surface of rGO, the tilt angle distribution appears less structured compared to the case of graphene. However, the dipole of the molecules in contact with the surface is smaller than the dipole of bulk water (see Table 1), similar to the case of graphene. This is because epoxides do not form strong hydrogen bonds with water; in fact, the hydrogen bond strength between a single water molecule and an epoxy group is estimated to be −0.20 eV, which is lower than the hydrogen bond strength in a water dimer, which is −0.22 eV (see SI). Consequently, interfacial water tends to preferentially form hydrogen bonds with surrounding water molecules rather than with surface epoxy groups. As mentioned in the previous paragraph, some epoxy groups transform into O^−^ groups, which are highly hydrophilic. The hydrogen bond between O^−^ and a single water molecule is calculated to be −0.43 eV. However, at low stoichiometries, the number of O^−^ groups is limited and therefore not sufficient to impart a hydrophilic character to the rGO surface.

**Table 1:** Average water molecule dipole in the monolayer first hydration layer.

material	water dipole
	(left/right) [D]
Graphene	2.7/2.8
GO 10 %ep 0 %hy	2.8/2.7
GO 0 %ep 10 %hy	2.8/2.9
GO 20 %ep 10 %hy	3.0/3.1
GO 10 %ep 20 %hy	3.1/3.2

As the degree of oxidation increases (30 % in Figure 3 panels d)–f)) and both epoxy and hydroxyl groups are present, the interface becomes more complex, making it challenging to understand the interplay between the effects of different functional groups. This complexity is further heightened by the transformation of some epoxide and hydroxyl groups into O^−^, imparting a partially negative charge to the surface (in agreement with experimental observations^[55]^). The overall effect is an increase in the hydrophilic character of the surface, as both −OH and O^−^ groups form strong hydrogen bonds with water (of −0.24 eV and −0.43 eV, respectively; see SI). The main indicators of increased surface hydrophilicity are the rise in water dipole moments for the molecules in contact with the rGO surface with increasing −OH content and the decrease in the height of the density peak close to the surface (see panel d) of Figure 3), as observed for other hydrophilic surfaces.^[56]^ Regarding the −OH tilt angle, an analysis of panel e) of Figure 3 shows that water molecules facing the rGO layer no longer exhibit −OH bonds perpendicular to the surface (no signal at high/low angles on the right/left side of the layer), as is typical of hydrophobic surfaces. The −OH preferential tilt angle encompasses a wide range of possibilities, varying from 15 to 90° on the right surface (50 to 140° on the left surface), as the optimization of hydrogen bonds with surface groups and other water molecules is possible for various molecular orientations.

In summary, when considering the wetting properties of rGO, we observed a much stronger hydrophilicity of rGO at higher oxidation levels, which we attribute to a coverage effect: as the overall oxidized area increases, the hydrophilic character of oxygen‐containing species prevails over the hydrophobic character of graphitic areas. From this investigation, we observed that O^−^ and −OH species form stronger hydrogen bonds with water than epoxides. Moreover, considering that O^−^ is frequently formed from −OH species, we can conclude that rGO layers with higher oxidation levels and a majority of −OH groups are the most hydrophilic.

### Water Confined in rGO Multilayer

**Figure 4:**
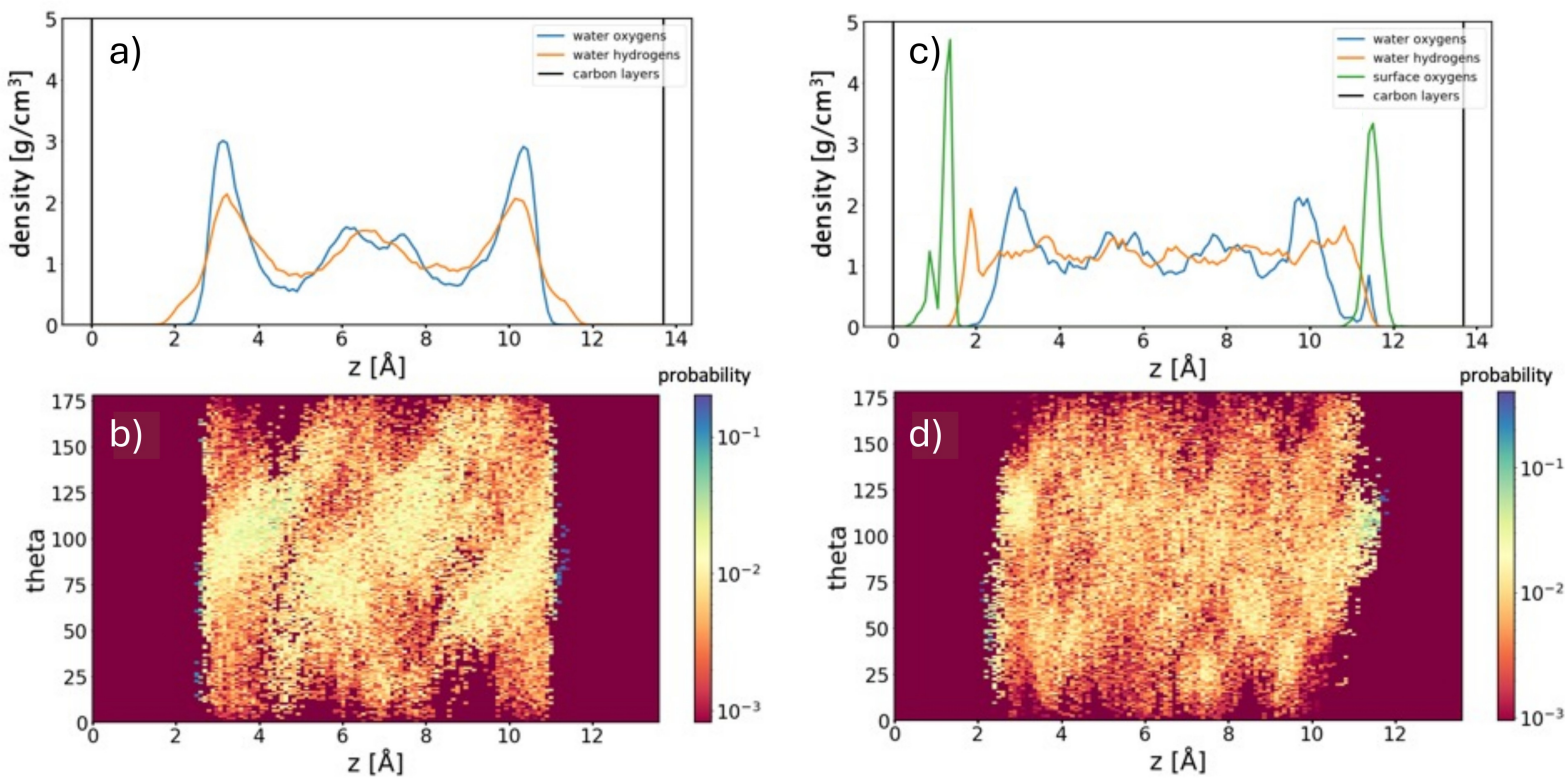
**Water confined between graphene layers with interlayer distance** ***d*** **=13.7** **Å** (left panels). Mass density distribution of oxygens and hydrogens in water, as a function of their distance from graphene layers (at 0 Å and 13.7 Å) (a). O−H bond orientation distribution of H_2_O molecules (b). **Water confined between rGO (10 % −O−20 % −OH) layers with interlayer distance** ***d*** **=13.7** **Å** (right panels). Mass density distribution of oxygens and hydrogens in water and mass density distribution of surface oxygens, as a function of their distance from the graphene layer (at 0 Å and 13.7 Å) (c). O−H bond orientation distribution of H_2_O molecules (d).

The analysis of water in multilayer rGO at an interlayer distance of 13.7 Å had the objective to investigate the influence of confinement on the hydration shell of the monolayer. An analysis of Figures 3 and 4 reveals that, independently on the monolayer stoichiometry, the hydration shell exhibits very similar features for both a single layer in water and a multilayer wetted structure. In other words, the structure of the interfacial water layer is independent of the distance between the monolayers. In particular, water overstructuring at the graphene surface is maintained, with the preferential orientation of −OH bonds confirming that one −OH bond points towards the graphene surface, while the other is nearly parallel to the surface. On the contrary, at the rGO surface, the situation is more complex, but the peak related to vertically oriented −OH is absent. Figure 4 also reveals that water density oscillations are slightly enhanced and more pronounced in the case of confined water systems with respect to isolated monolayer systems. Moreover, because of the enhanced oscillations in our confined structures water never fully recovers flat bulk density.

To investigate the effects of confinement on the diffusivity of water molecules, we computed their average self‐diffusion coefficient, as detailed in the Method section, comparing the case of graphene and of rGO samples at different stoichiometries with the self‐diffusion coefficient computed by Gygi and collaborators^[57]^ for bulk water (and with standard deviation ). From literature we expected water confined between graphene layers to have a very high diffusivity,[58] but surprisingly we computed a lateral self‐diffusion coefficient which is comparable to that of bulk water. In Table 2 are reported the average self‐diffusion coefficients computed for water molecules confined between graphene and rGO layers. While vertical diffusivity () is severely quenched with respect to bulk water in all cases, as expected since this direction is confined, lateral diffusivities () are comparable between graphene and rGO. Although slight lateral diffusivity reductions (~20 %) can be appreciated increasing the oxidation level of rGO from 10 % coverage to 30 %, these values are still comparable to that of water confined between graphene flakes and of bulk water. These results are therefore in conflict with some previous studies which assumed water flowing through multilayer rGO membranes to have a very high diffusivity in correspondence of hydrophobic graphene areas and a very slow diffusivity where hydrophilic oxidized clusters are present.[^58^, ^59^, ^60^] Our study, in agreement with a paper from Cho and collaborators,^[61]^ demonstrates that the diffusivity of water molecules in fully hydrated rGO membranes is not impeded by the presence of hydrophilic oxygen‐containing species.

**Table 2:** Self‐diffusion coefficient of confined water molecules.

material	*D_xy_*	*D_z_*
	[10^−5^ cm^2^/s]	[10^−5^ cm^2^/s]
Graphene	2.8	1.0
GO 10 %ep 0 %hy	2.3	0.8
GO 0 %ep 10 %hy	3.8	1.1
GO 20 %ep 10 %hy	1.9	0.5
GO 10 %ep 20 %hy	1.8	0.6

## Conclusions

In this work, we investigated the interactions between rGO at different stoichiometries and liquid water, with a focus on properties relevant to multilayer membranes. By analyzing long Car‐Parrinello MD trajectories, representing interfaces between single rGO monolayers and water, we studied the role of oxygen‐containing species in determining rGO′s hydrophilicity. Specifically, we considered the net orientation of interfacial water molecules and their average electrostatic dipole as reliable indicators of surface wettability. From this analysis, we concluded that the hydrophilicity of rGO is directly proportional to its oxidation level, as evidenced by the stronger hydrogen bond networks formed by water molecules near rGO monolayers with higher oxidation coverage. This conclusion aligns with earlier experimental findings, such as those by Lian et al., who demonstrated that oxygen‐containing functional groups significantly increase water uptake and adsorption, highlighting that highly oxidized graphene materials exhibit greater hydrophilicity and stronger water interactions.^[62]^ To better assess the contribution of specific oxygen‐containing species to the wettability of this material, we investigated the strength of hydrogen bonds formed between water molecules and individual species adsorbed on a graphene layer. We found that −OH and O^−^ groups interact more strongly with water than epoxides and ethers, leading us to conclude that higher concentrations of these groups result in highly hydrophilic rGO monolayers. Furthermore, we observed that the composition of rGO flakes is metastable. This metastability was evident in the evolution of −OH and epoxide groups into negatively charged O^−^ species and the desorption of −OH groups. This process plays a significant role in rGO′s interaction with water and is consistent with previous studies showing how oxygen‐containing groups evolve when in contact with water molecules.^[37]^

In our study of water behavior confined between rGO layers, we provided evidence that the self‐diffusion coefficient of water molecules confined between rGO flakes is comparable to that of bulk water. These findings, contrary to some earlier studies,[^58^, ^59^, ^60^] suggest that water transport in rGO multilayer membranes is practically uniform across the entire samples, with only minor differences between pristine graphitic regions and oxidized areas. This contrasts with the findings reported in Ref. [63], where it was proposed that the formation of hexagonal ice bilayers between graphene oxide layers is crucial for enabling anomalous water permeation through graphene oxide membranes. Our results indicate that uniform water permeability can occur without the formation of such ice‐like structures, which are unlikely to form. Indeed, we observed no evidence of such structured ice formations, confirming instead that water permeates easily through rGO membranes keeping a liquid structure. This reinforces the material′s potential for applications in filtration and environmental remediation. Our conclusions are more closely aligned with the experimental work, which demonstrated the facile permeation of water through graphene‐based membranes without the need for ice‐like structures formation.^[58]^

## Conflict of Interests

Please enter any conflict of interest to declare.

1

## Supporting information

As a service to our authors and readers, this journal provides supporting information supplied by the authors. Such materials are peer reviewed and may be re‐organized for online delivery, but are not copy‐edited or typeset. Technical support issues arising from supporting information (other than missing files) should be addressed to the authors.

Supporting Information

## Data Availability

The data that support the findings of this study are available from the corresponding author upon reasonable request.
