# Supplementary material for: Insights into the Hydration Layer of Reduced Graphene Oxides: A Computational Study
Source: ChemSusChem. 2024 Nov 17;18(4):e202400520. doi: 10.1002/cssc.202400520 (PMC11826141; doi:10.1002/cssc.202400520)
Supplement: Supplementary file 1 — Supporting Information [file CSSC-18-e202400520-s001.pdf]

ChemSusChem

Supporting Information

# Insights into the Hydration Layer of Reduced Graphene Oxides: A Computational Study

Filippo Savazzi, Francesca Risplendi, and Giancarlo Cicero*

# Insights into Hydration Layer of Reduced Graphene Oxides: A Computational Study

**Supporting Information**

Filippo Savazzi,[a] Francesca Risplendi,[a] Giancarlo Cicero,*[a]

## Interactions between rGO and water

Understanding the interaction between these specific oxygen-containing groups and water molecules is fundamental to investigate their influence on surface wettability, therefore we created additional model structures containing only the specific isolated oxygen-containing species and one or two water molecules. These structures were used to estimate the strength of hydrogen bonds (H-bonds) between water molecules and oxygen-containing groups in rGO, by means of accurate DFT simulations (details for these calculations are reported in Section Method of the manuscript). For epoxide (Figure S1 A), 1,2-ether (Figure S1 B) and hydroxyl (Figure S1 D and E) groups we computed the H-bond energy as the difference between the energy of the relaxed rGO sample with one water molecule ($E_{sys}^{H_2O}$), the energy of the relaxed rGO sample without water ($E_{sys}$) and the energy of a water molecule ($E_{mol}^{H_2O}$):

$$E_{H-bond} = E_{sys}^{H_2O} - E_{sys} - E_{mol}^{H_2O} \tag{1}$$

In the case of an hydroxyl species, this can either be an H-bond donor (Figure S1 D) or acceptor (Figure S1 E). $O^-$ groups (Figure S1 C) on the other side, only exist in presence of water and indeed we discovered performing DFT relaxations that they need at least two water molecules to stabilize at ground state (with one water molecule, epoxides are favored). For this reason we computed the H-bond energy between an $O^-$ species and a single water molecules as:

$$E_{H-bond} = \frac{E_{sys}^{H_2O} - E_{sys,constr} - 2E_{mol}^{H_2O}}{2} \tag{2}$$

where $E_{sys,constr}$ is the energy of a rGO system without water, where the oxygen atom was constrained to remain vertical during DFT relaxation.

From our calculations, the H-bond energy between an epoxide species and a water molecule at ground state is $E_{H-bond}^{epo} = -0.20$ eV, slightly lower than the H-bond energy of a water dimer computed at the same conditions ($E_{H-bond}^{H_2O} = -0.22$ eV). The relaxed distance between the epoxide and the oxygen in the water molecules is $d_{O-O} = 2.90$ Å, the distance between the epoxide and the hydrogen in the water molecule is $d_{O-H} = 1.93$ Å, while the distance between the oxygen in the water molecule and the graphitic plane is $d_{O-C} = 3.39$ Å, as reported in Figure S1 A.

For the interaction between a 1,2-ether species and a water molecule, we computed a H-bond energy $E_{H-bond}^{eth} = -0.14$ eV which is lower than the H-bond energy between water and an epoxide species, indicating a weaker interaction of water with 1,2-ether groups in rGO.

In the case of $O^-$ groups, we computed a H-bond energy between this species and one water molecule $E_{H-bond}^{O^-} = -0.43$ eV which instead is considerably higher than the H-bond energy of a water dimer ($E_{H-bond}^{H_2O} = -0.22$ eV), highlighting a very strong affinity between water and $O^-$ groups in rGO. After relaxation to ground state energy, the two water molecules are positioned with their hydrogen atoms in proximity to the $O^-$ in a configuration which is almost perfectly symmetrical. The distance between the $O^-$ and the oxygen in one water molecules is $d_{O-O} = 2.71$ Å, the distance between the O- and the hydrogen in one water molecule is $d_{O-H} = 1.71$ Å, while the distance between the oxygen in the water molecule and the graphitic plane is $d_{O-C} = 3.10$ Å, as reported in Figure S1 C.

Finally, for hydroxyl species where the oxygen shares its lone pair with a water molecule (donor –OH, Figure S1 D) we computed a H-bond energy $E_{H-bond}^{-OH,don} = -0.24$ eV, whereas for acceptor hydroxyl species (acceptor –OH, Figure S1 E) we computed a H-bond energy $E_{H-bond}^{-OH,acc} = -0.23$ eV. These energies are comparable, showing that

in either configurations –OH groups have similar interactions with water, as H-bond energies are comparable with that between two water molecules ($E^{H_2O}_{H-bond} = -0.22$ eV), highlighting a good affinity between water and –OH groups in rGO.

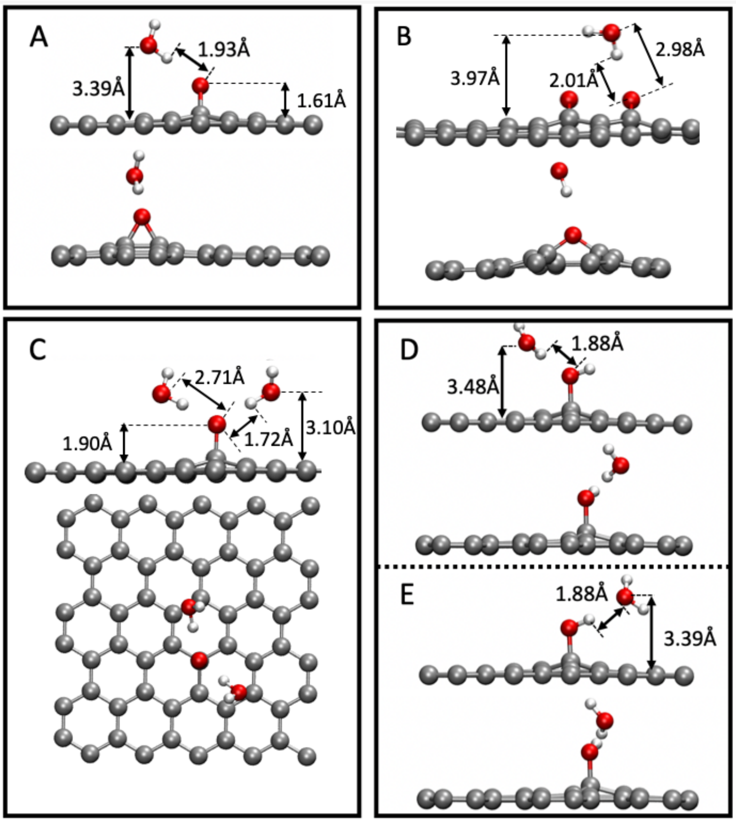


**Figure S1.** Relaxed structures modeling the interaction between (A) an epoxide group and a water molecule, B) two 1,2-ethers and a water molecule, C) one $O^-$ and two water molecules, D) one hydroxyl group (donor) sharing its lone pairs with a water molecule and E) one hydroxyl (acceptor) interacting with lone pair from a water molecule.

## Water Spatial Distribution Function

To qualitatively assess the arrangement of the water molecules in the first hydration shell of the -OH and -O- functional groups present on the rGO surface, we calculated the Spatial Distribution Function (SDF) around these moieties using the TRAVIS code[1,2].

As reported in Figure S2, the SDF analysis reveals that water is more structured around the -OH group. It is apparent that on average the -OH group donate one H-bond to a solvent water molecule, as demonstrated by the presence of the red isosurface structure right in front of the hydroxyl H atom (panel a of Figure S2). At the same time, on average the hydroxyl group accepts twos H-bonds acceptor from two water molecules in the solvent, as shown by the two distinctive blue isosurface features on the side of the oxygen atom of the hydroxyl group (panel b of Figure S2). These features are related to water hydrogen atoms interacting with the lone pairs of the surface -OH. On the contrary the isosurface density plot is less structures around the -O- group. In this case there is a more delocalized blue isosurface feature (panel c of Figure S2) at the rGO/water interface around the -O- group. This is related to the water H-atoms interacting with the -O- which function as H-bond acceptor only (the epoxide group cannot donate H-bonds). As discussed in the previous paragraph, in the case -O- the strength of the hydrogen bond with water is weaker that the water/water H-bonds and hydroxyl/water H-bond interaction and this results in a less structured SDF.

## References

[1] M. Brehm, M. Thomas, S. Gehrke, B. Kirchner, *The Journal of Chemical Physics* **2020**, *152*, 164105.
[2] M. Brehm, B. Kirchner, *Journal of Chemical Information and Modeling* **2011**, *51*, 2007, pMID: 21761915.

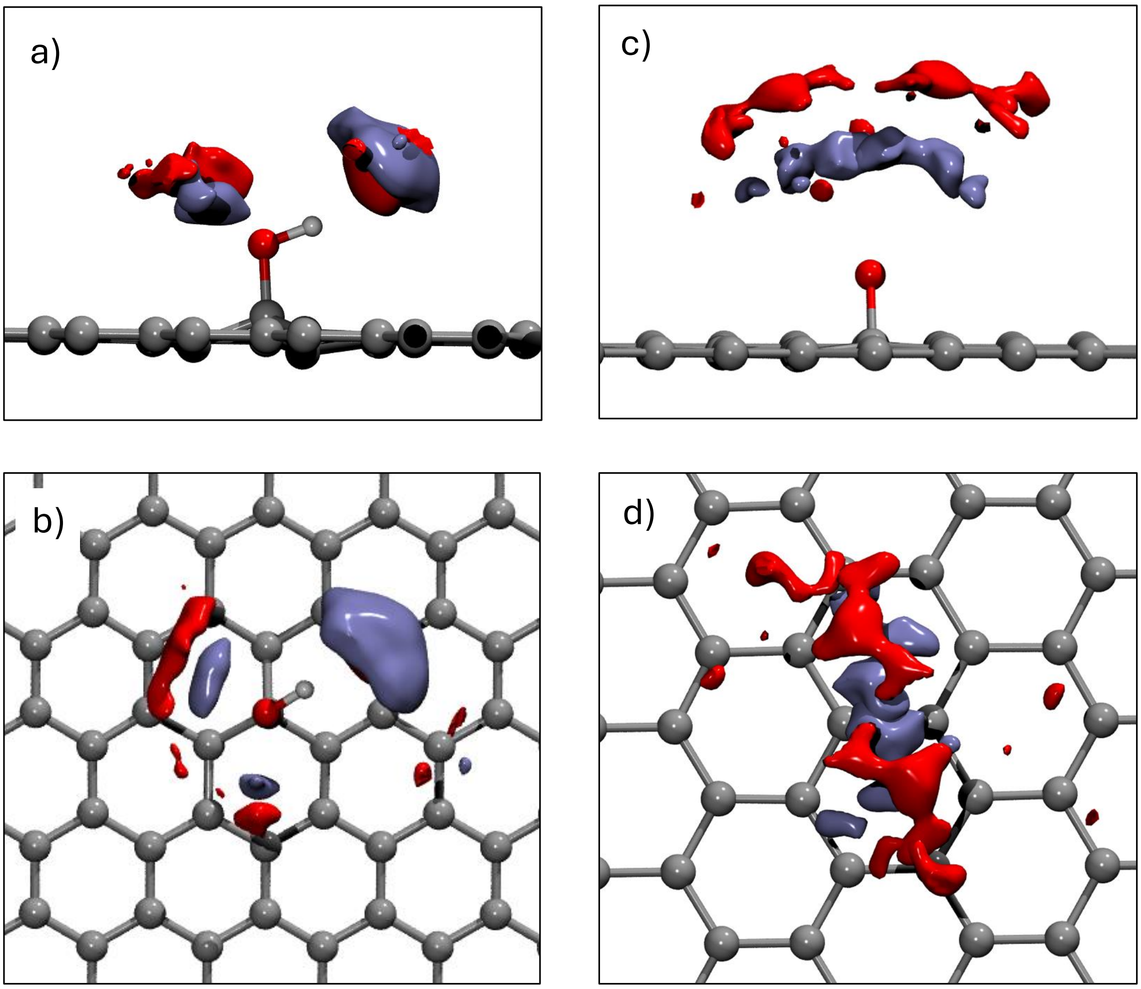


**Figure S2.** Spatial Distribution Functions (SDF) for hydrogen (blue isosurface) and oxygen (red isosurface) atoms of the water molecules around hydroxide and epoxide functional groups. Panels (a) and (b) represent the side and top views of the SDF around the -OH group, respectively, while panels (c) and (d) depict the side and top views for the SDF around the epoxide. The isosurfaces are set at 40% of the maximum value.
